# Multitargeted Internal Calibration for the Quantification of Chronic Kidney Disease-Related Endogenous Metabolites Using Liquid Chromatography–Mass Spectrometry

**DOI:** 10.1021/acs.analchem.3c02069

**Published:** 2023-09-01

**Authors:** Gioele Visconti, Miguel de Figueiredo, Oriane Strassel, Julien Boccard, Nicolas Vuilleumier, David Jaques, Belén Ponte, Serge Rudaz

**Affiliations:** † School of Pharmaceutical Sciences, University of Geneva, CMU − Rue Michel-Servet 1, 1211 Geneva 4, Switzerland; ‡ Institute of Pharmaceutical Sciences of Western Switzerland, University of Geneva, CMU − Rue Michel-Servet 1, 1211 Geneva 4, Switzerland; § Department of Genetic and Laboratory Medicine, Geneva University Hospitals (HUG), Rue Gabrielle-Perret-Gentil 4, 1205 Geneva, Switzerland; ∥ Service of Nephrology, Geneva University Hospitals (HUG), Rue Gabrielle-Perret-Gentil 4, 1205 Geneva, Switzerland

## Abstract

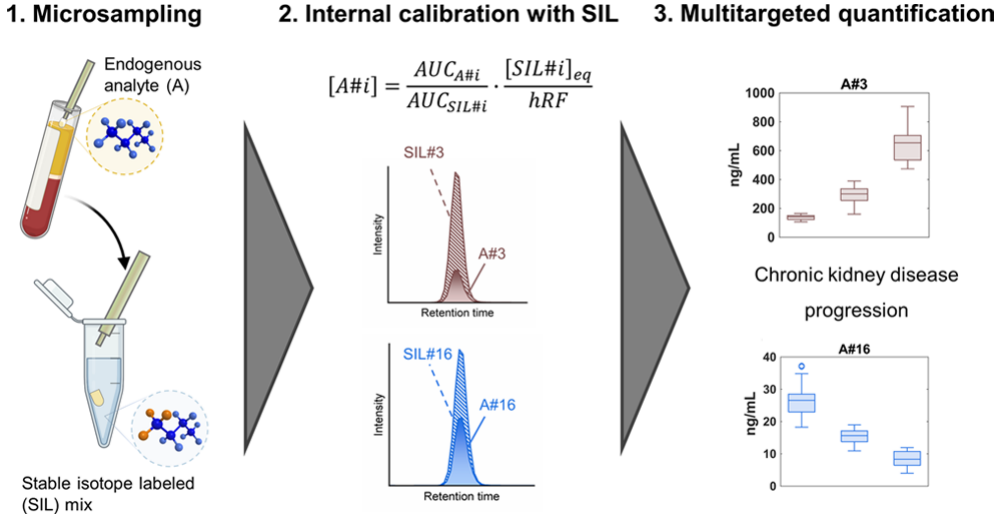

Accurate quantitative analysis in liquid chromatography–mass spectrometry (LC–MS) benefits from calibration curves generated in the same matrix as the study sample. In the case of endogenous compound quantification, as no blank matrix exists, the multitargeted internal calibration (MTIC) is an attractive and straightforward approach to avoid the need for extensive matrix similarity evaluation. Its principle is to take advantage of stable isotope labeled (SIL) standards as internal calibrants to simultaneously quantify authentic analytes using a within sample calibration. An MTIC workflow was developed for the simultaneous quantification of metabolites related to chronic kidney disease (CKD) using a volumetric microsampling device to collect 20 μL of serum or plasma, followed by a single-step extraction with acetonitrile/water and liquid chromatography–tandem mass spectrometry (LC–MS/MS) analysis. Since a single concentration of internal calibrant is necessary to calculate the study sample concentration, the instrument response function was investigated to determine the best SIL concentration. After validation, the trueness of 16 endogenous analytes in authentic human serum ranged from 72.2 to 116.0%, the repeatability from 1.9 to 11.3%, and the intermediate precision ranged overall from 2.1 to 15.4%. The proposed approach was applied to plasma samples collected from healthy control participants and two patient groups diagnosed with CKD. Results confirmed substantial concentration differences between groups for several analytes, including indoxyl sulfate and cortisone, as well as metabolite enrichment in the kynurenine and indole pathways. Multitargeted methodologies represent a major step toward rapid and straightforward LC–MS/MS absolute quantification of endogenous biomarkers, which could change the paradigm of MS use in clinical laboratories.

## Introduction

Chronic kidney disease (CKD) is a growing health concern affecting millions of people worldwide, partly due to population aging.^1^ CKD is characterized by progressive damages to kidneys that impair their ability to function efficiently, leading to a range of complications including anemia, metabolic disorders, cardiovascular disease, and end-stage renal disease.^2^ CKD severity is classified into five stages based on the estimated glomerular filtration rate (eGFR) calculated from serum creatinine and three additional stages are also determined by albuminuria levels.^3^ However, the lack of specificity and sensitivity of current biomarkers for CKD may lead to unnecessary expensive testing, or a delay in receiving medical care.^4^ As current treatments are mainly aimed at preventing CKD progression and mitigating associated complications, early detection and intervention are necessary to improve prognosis.

Liquid chromatography–mass spectrometry (LC–MS) represents a powerful and widely used analytical approach for the determination of biochemical alterations of various pathophysiological processes.^5−7^ Comprehensive metabolome analysis using LC–MS untargeted workflows has recently emerged as a promising approach in clinical research.^8^ In such a context, untargeted metabolomics often aims to identify putative biomarkers by comparing relative metabolic signatures between control and case-study cohorts.^9^ In most cases, untargeted metabolomics does not provide any information about metabolite concentrations, as only analyte intensity ratios between the control and case-study cohorts are considered.^9^ This is mainly due to the wide variety of physicochemical properties and metabolite concentrations ranging from pg/mL to mg/mL.^10^ Targeted metabolomics can then be implemented as a subsequent approach by focusing on a specific analyte subset. In this context, quantification methods have to be developed to translate MS-measured signals into concentrations, allowing for clinical interpretation and comparison between studies.^11^

Recent untargeted metabolomic studies have highlighted potential biomarkers whose plasma levels correlate with CKD progression. Those are mostly related to the tryptophan pathway, the synthesis of mineralocorticoids and some modified amino acids.^12,13^ However, quantification of targeted analytes is needed to validate the selection of relevant metabolites by confirming prior results and provide effective diagnostic values for clinical routine.

A key challenge in endogenous metabolite quantification by LC–MS is the absence of a true blank matrix (*i.e.*, a matrix without the analyte of interest) to build the calibration curve, thus hampering reliable quantification in terms of accuracy and precision.^14^ Although recent bioanalytical guidelines started a formal discussion on this subject, a consensus on the blank matrix is missing.^15,16^ In the context of CKD-related metabolites, Whiley et al. proposed a multitargeted approach for the quantification of 18 analytes in serum and plasma by performing a calibration curve in neat solution.^17^ Even if the extraction recovery and the matrix effect were corrected with deuterated internal standards, as recommended by international bioanalytical guidelines, this calibration approach bears limitations.^18^ The conventional workflow involves a full external calibration curve for each batch of samples, leading to a significant increase in both cost per batch and time required for instrument turnaround.^19^ Even though the usefulness of including a calibration curve in each batch has been debated, most clinical laboratories still operate in this fashion.^20^ To improve laboratory efficiency and make endogenous metabolites’ analysis more accessible and attractive in a clinical environment, alternative calibration methodologies have been proposed. The most recent strategies include reducing the number of calibrants or restricting internal calibration to a limited number of analytes.^21−23^

Relying on previous findings regarding biomarker of CKD progression, the aim of this work was to develop a fit-for-purpose LC–MS/MS method to simultaneously quantify a relatively large panel of biomarkers from different biological pathways using internal calibration (IC). The proposed strategy includes an easy and accurate volumetric microsampling collection (20 μL) from plasma tubes without the use of micropipettes, thus facilitating its implementation in clinical routine. The method was validated in terms of repeatability, intermediate precision, and trueness for 16 metabolites and subsequently applied to human plasma samples from a clinical cohort to define biological intervals. CKD progression in patients was evidenced at different stages of the disease, thanks to absolute quantification of the panel of biomarkers, with a clear discrimination toward healthy individuals.

## Materials and Methods

### Chemicals and Reagents

11-Deoxycortisol, 11-deoxycortisol-2,3,4-^13^C_3_, 5-hydroxyindole-3-acetic acid, 5-hydroxyindole-3-acetic-4,6,7-d_3_-acid-d_2_, anthranilic acid, ascorbic acid, corticosterone, corticosterone-9,11,12,12-d_4_, cortisol, cortisol-2,3,4-^13^C_3_, cortisone, cortisone-2,3,4-^13^C_3_, dehydroepiandrosterone 3-sulfate, 3-hydroxykynurenine, indoxyl sulfate, kynurenic acid, tryptophan, nicotinamide, and phenylacetyl-glutamine were purchased from Sigma-Aldrich (Buchs, Switzerland). Androsterone 3α-glucuronide was provided from National Analytical Reference Laboratory (NARL, Sydney, Australia) while androsterone-2,2,4,4-d_4_ 3α-glucuronide and dehydroepiandrosterone-3-2,2,3,4,4-d_5_ sulfate were supplied from Cerilliant (Round Rock, TX, USA). *N*-Acetyl-leucine was purchased from Acros Organics (Geel, Belgium) while 3-hydroxyanthranilic-4,5,6-d_3_ acid, *n*-acetyl-alanine, *n*-acetyl-alanine-3,3,3-d_3_, and phenyl-d5-acetyl-glutamine were provided by Toronto Research Chemicals (TRC, Toronto, ON, USA). Indole-3-acetic acid, kynurenine, and serotonin-1,1,2,2-d_4_ were obtained from Alfa Aesar (Kandel, Germany). 3-Hydroxyanthranilic acid, kynurenic acid-3,5,6,7,8-d_5_, and serotonin-1,1,2,2-d_4_ were provided by Cayman Chemical (Ann Arbor, Michigan, USA). Kynurenine-1,2,3,4,5,6-^13^C_6_, indoxyl sulfate-1,2,3,4,5,6-^13^C_6_, indole-3-acetic-1,2,3,4,5,6-^13^C_6_ acid, and *n*-acetyl-leucine-1,1,1,2,2,2,3,4,4,5-d_10_ were supplied by Alsachim (Illkirch, France). Tryptophan-1,3,4,5,6,7,8,9,10,11,13-^13^C_11_, 5-hydroxy-indole-3-acetic acid-3,4,5,6,7,7-^13^C_6_, 3-hydroxykynurenine-1,2,3-^13^C_3_,^15^N, anthranilic-4,5,6,7,8,9-^13^C_6_ acid, and nicotinamide-1,2,3,4,6,7-^13^C_6_ were provided by Cambridge Isotope Laboratories (CIL, Andover, Massachusetts, USA).

Stock solutions of each analyte and stable isotope labeled (SIL) standard were prepared at a concentration of 1 mg/mL or 100 μg/mL, depending on the availability of standards. Most of the chemical standards were dissolved in methanol/water (1:1 v/v) with 10 mM ascorbic acid. When necessary, 0.05 M ammonium hydroxide or 0.1 M formic acid were added to increase solubility. Standards were dissolved in pure methanol or dimethyl sulfoxide, when not soluble in a hydroalcoholic solution. Table S1 summarizes the solvents used to dissolve each chemical standard, as well as the chemical purities and SIL isotopic enrichment. Intermediate solutions with appropriate concentrations were prepared by successive dilution of the stock solutions in methanol/water (1:1 v/v). Concentrations of SIL stock solutions were carefully evaluated through individual injections in the ultra-high performance liquid chromatography (UHPLC) system and measuring the ultraviolet response. The obtained signal was then compared to the same analysis of the corresponding unlabeled analogue solution that was gravimetrically prepared or commercially certified.

UHPLC–MS-grade formic acid was obtained from Biosolve (Valkenswaard, The Netherlands). Sodium hydroxide solution and ammonium fluoride (chemical purity >99.9%) were purchased from Sigma-Aldrich. UPLC–MS-grade acetonitrile, water, and methanol were obtained from Fisher Scientific (Loughborough, UK). Volumetric absorptive microsampling (VAMS) devices were purchased from Neoteryx (Torrance, USA).

### Calibrants and Quality Control Samples

**Figure 1:**
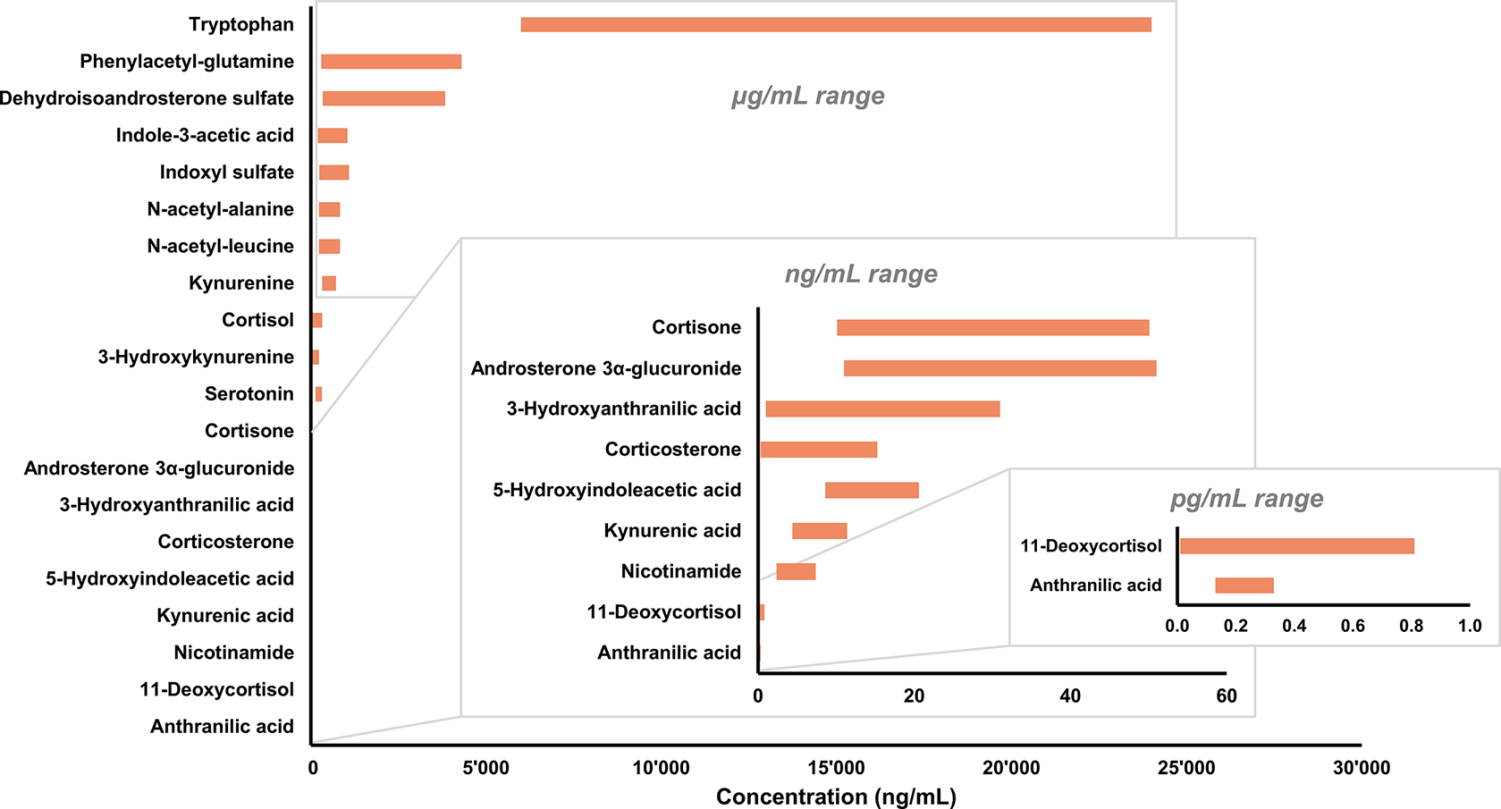
Representation of the biological reference interval that needs to be covered based on values from the public accessible Human Metabolome Database.^10^

The MTIC in biological study samples was performed with a SIL mix prepared in a protein precipitant solution, namely, acetonitrile/water (9:1 v/v). The concentration to be used for each SIL was determined through different instrument response linearity assays. Nine analyte working solutions (cal 1 to 9) and six SIL working solutions (SIL 5 to 9) were prepared in pure H2O (Tables S2 and S3) based on the expected biological concentration range (Figure 1). For the six SIL investigated levels, a nine-point calibration curve was generated by introducing the corresponding SIL working solution. The selected SIL composition is detailed in Table S4.

In-house serum and plasma quality controls (QCs) were created by pooling three healthy male samples and three healthy female samples. Samples were provided by the Geneva University Hospital with written consent from the donors. Absolute endogenous concentrations were determined using the standard addition method. These concentrations were then fortified with aqueous mixes containing the authentic analytes, resulting in additional QC samples with higher target values (Table S5). For intra- and inter-assay accuracy and precision, the pooled serum was diluted in charcoal dextran single stripped pooled human serum (Dunn Labortechnik GmbH, Asbach, Germany) to obtain QC samples with lower concentration values (Table S6). All pipetting and liquid handling steps for preparing QC samples, intermediate and working solutions were performed using a semi-automated electronic pipetting workflow (Andrew Alliance Pipette+, Waters Corporation, Daettwil, Switzerland).

All biological QC samples, stock, intermediate, and working solutions were maintained at −80 °C in 1.5 mL Eppendorf Safe-Lock Tubes (Eppendorf, Hamburg, Germany).

### Sample Preparation

For both serum and plasma, 20 μL was collected directly from blood sampling tubes with volumetric absorptive microsampling (VAMS) devices from Neoteryx (Torrance, CA, USA) and allowed to dry for three hours before extraction. The tips of VAMS devices, attached to the plastic handler, were removed and placed into Eppendorf tubes. On-tip protein precipitation and metabolite extraction were carried out with 200 μL of acetonitrile/water (9:1 v/v) containing SILs. Samples were sonicated for 15 min and the extraction solvent was transferred to a polypropylene vial and dried for 90 min in a Savant 210A SpeedVac (Thermo GmbH, Langenselbold, Germany). The dried samples were reconstituted in 20 μL of methanol/water (5:95 v/v) and subsequently analyzed with UHPLC–MS/MS.

### UHPLC–MS/MS Analysis

An Acquity H-Class (Waters Corporation, Milford, MA, USA) UHPLC system equipped with a quaternary solvent delivery pump, a flow-through-needle autosampler, a column oven, and a photodiode detector was used with a Kinetex Biphenyl column (150 × 2.1 mm, 1.7 μm; Phenomenex, California, USA). Mobile phase A was pure water +0.1% formic acid, and mobile phase B was pure methanol +0.1% formic acid. The analytical flow rate was set at 0.3 mL/min, and the column temperature was 45 °C. Post-column infusion of 20 mM ammonium fluoride in methanol was combined at a flow rate of 5 μL/min using the fluidics system on the mass spectrometer under full software control. Acetonitrile/water (50:50) was used for needle wash. The gradient started from 2% B and increased linearly to 100% over 14 min, followed by 3 min at 100% B and 9 min under the initial conditions (2% B), for a total run time of 26 min. The injection volume was 10 μL and samples were kept at 6 °C in the autosampler.

A Xevo TQ-XS triple quadrupole mass spectrometer (Waters Corporation, Milford, MA, USA) equipped with an electrospray ion source (ZSpray) operated in polarity switching ion mode and acquiring data in scheduled multiple reaction monitoring (MRM) was used. At least two transitions of the main isotopic form for both analyte and SIL were selected for quantification and confirmation of the metabolites (Table S7). Cone voltages and collision energies were individually optimized using Intellistart (Waters Corporation, Milford, MA, USA). Each metabolite standard was infused using a mixture of methanol/water (60:40 v/v) containing 0.1% formic acid and 0.2 mM ammonium fluoride at a flow rate of 0.3 mL/min. Depending on the ionization of each compound, 1 or 10 μg/mL solutions of each standard in methanol/water (50:50 v/v) were infused at a flow rate of 10 μL/min. Ion source conditions were optimized by replicate injections of a standard mix, and by modifying the ionization parameters to obtain the highest sensitivity. Capillary voltage was maintained at 0.7 kV in positive and negative ionization modes, source temperature was 150 °C, the desolvation temperature and gas flow were 650 °C and 800 L/h, respectively, and the cone gas was 150 L/h. Dwell time was set to 10 ms for each transition except for dehydroisoandrosterone sulfate, which was 5 ms. To reduce contamination in the mass spectrometer and ensure signal stability over batch runs, the UHPLC flow rate was diverted in waste at the beginning of injection (0–1.9 min) and after the gradient (15–26 min). MassLynx 4.2 software (Waters Corporation, Milford, MA, USA) was used for systems control and optimization of mass transitions.

Skyline MS 22.2 (MacCoss Lab Software, Virginia, USA) was used for peak area integration,^24^ and the computation of sample concentrations was performed with Python 3.9 using an automated in-house workflow.

### Patient Samples

Participating groups involved nineteen control plasma samples from healthy volunteers, sixteen CKD participants followed at the Geneva University Hospitals ambulatory nephrology consultation with an eGFR of 44–15 mL/min/1.73 m^2^ (CKD3b/4), and sixteen patients undergoing hemodialysis 3 times per week (CKD5). The study was approved by the Human Research Ethics Committee of the Canton of Geneva in Switzerland (amendment to the study PB_2016-02160 CER-13125), and all participants signed an informed consent form. For all patients and healthy volunteers, plasma samples were collected in ethylene diamine tetra acetic acid dipotassium tubes (EDTA-K_2_, Vacutainer Hemogard, Becton Dickinson, NJ, USA). In CKD5 patients, blood sample was taken immediately prior to the mid-week hemodialysis session. The samples were then analyzed using the developed LC–MS/MS method to quantify endogenous metabolites that were found to be correlated with CKD progression.^12^ Creatinine concentration was measured with standardized kinetic colorimetric Jaffé reaction using a Cobas 8000 c702 (Roche Diagnostics, Rotkreuz, Germany) at Geneva University Hospitals.

### Internal Calibration

The SIL-mix was spiked directly into the biological samples for metabolite quantification and results were computed using an automated in-house workflow, as described elsewhere.^23,25^ Briefly, the following equation was used:
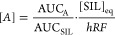
1where [*A*] defines the concentration of analyte; AUC_A_ and AUC_SIL_ are the integrated areas of the analyte and stable isotope-labeled analogue, respectively; *hRF* is the historical response factor;^20^ and [SIL]_eq_ is the SIL-analyte concentration equivalent.^23^

The computed RFs for all analytes are summarized in Table S8.

### Intra- and Inter-Assay Accuracy and Precision

The repeatability, intermediate precision, and trueness were obtained with a set of QCs at six concentration (*k* = 6) spiked in human serum and extracted in quadruplicate (*n* = 4) over four validation assays (*j* = 4). The intraday precision (repeatability) and intermediate precision were expressed as a coefficient of variation (CV) after nested analysis of variance (ANOVA) decomposition.^26,27^ Trueness was calculated as the average of relative bias over the four validation assays. The method was considered accurate and precise if CV values were below 20% and accuracies were in the range of 70–130%.^28−30^

### Statistical Analysis and Visualization

Prism version 9.1.4 (GraphPad Software, California, USA) was used for analysis of covariance to statistically compare the slopes obtained for each analyte-SIL couple and histograms creation. Box plot and principal component analysis were performed using MatLab R2022a (MathWorks Inc., Natick, MA, USA). UHPLC chromatogram was created using Origin Pro 2022 version 9.9 (OriginLab Corporation, Northampton, MA, USA).

## Results and Discussion

An efficient multitargeted internal calibration (MTIC) suitable for the absolute quantification of multiple metabolites related to CKD was developed. Metabolite selection was based on published literature evidencing stratification of CKD severity considering specific biological pathways, such as kynurenine or indole acid biosynthesis.^31,32^ However, no information about absolute concentrations was available because only relative signal intensities were compared between control and CKD groups. The quantification of endogenous metabolites in complex biological samples such as serum and plasma represents a real analytical challenge due to potential matrix effects.^18^ To overcome the lack of a true blank matrix to construct the calibration curve, direct quantification was performed in the native sample based on the analyte-to-SIL response ratio and the SIL spiked amount (eq 1).^20^ The developed and validated MTIC assay was considered to be acceptable for application to clinical samples. Since sample collection was performed in a hospital setting, the workflow has been adapted to accommodate both hospital operational constraints and sample preparation reproducibility. Accurate SIL spiking requires qualified personnel to store and handle SIL mixtures, which is also time-consuming and incompatible with patient flows in hospitals. Consequently, SILs were added at the sample preparation step, where VAMS devices were used to collect volumetric plasma directly from Vacutainer tubes prior to sending them for routine clinical analysis and shipping the VAMS devices for LC–MS/MS analysis. This approach was applied to clinical study samples and allowed the classification of chronic kidney disease progression based on the obtained concentration values for the selected metabolite panel.

### Chromatography and Mass Spectrometry

**Figure 2:**
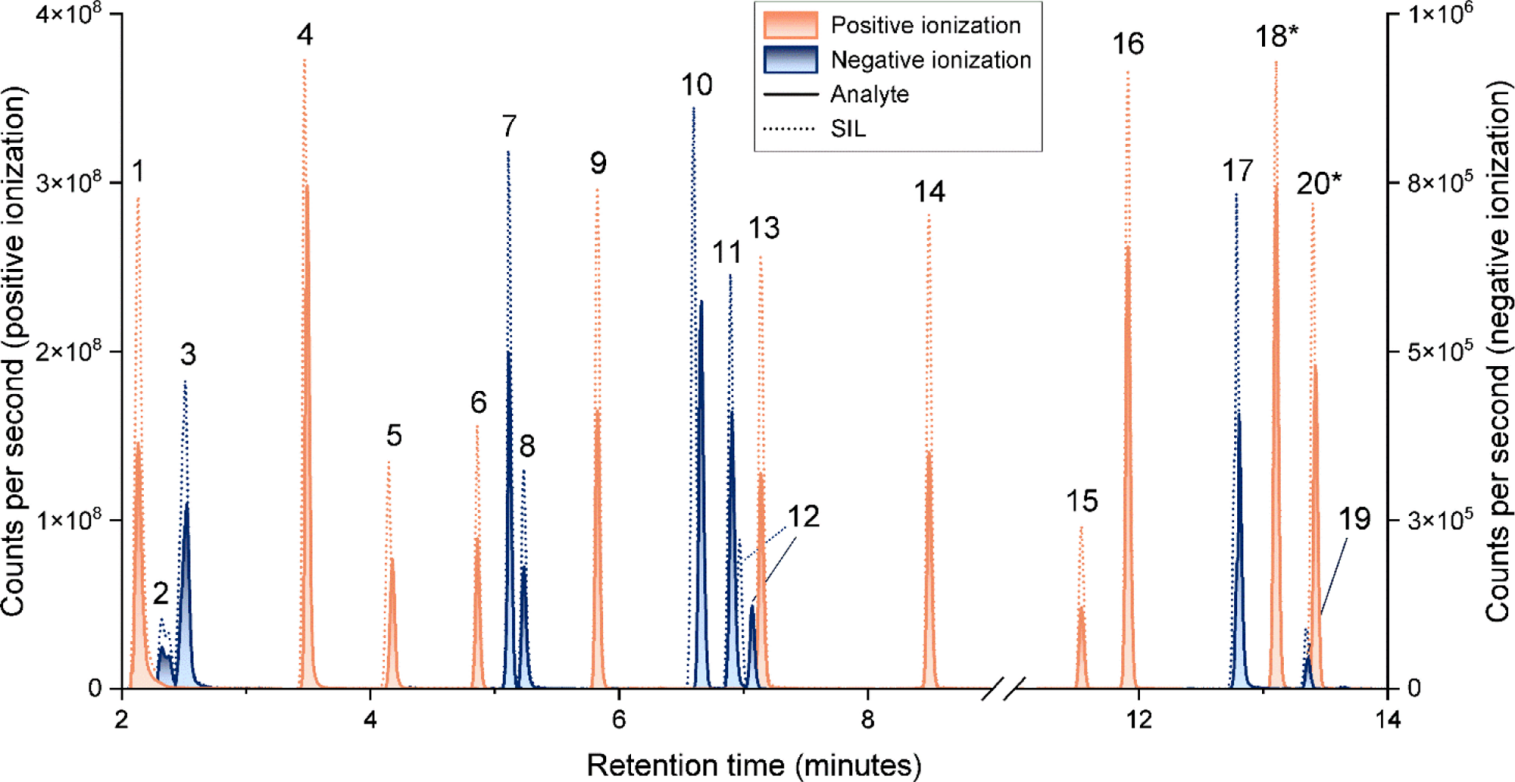
Representative chromatogram of analytes and SIL analogues (in italic): (1) nicotinamide(-^13^C_6_); (2) 3-hydroxykynurenine(-*^13^C_3_*, *^15^N*); (3) *n*-acetyl-alanine(-*d_3_*); (4) serotonin(-*d_4_*); (5) 3-hydroxyanthranilic acid(-*d_3_*); (6) kynurenine(-*^13^C_6_*); (7) tryptophan(-*^13^C_11_*); (8) indoxyl sulfate(-*^13^C_6_*); (9) 5-hydroxyindoleacetic acid(-*^13^C_6_*); (10) phenylacetyl-glutamine(-*d_5_*); (11) kynurenic acid(-*d_5_*); (12) *n*-acetyl-leucine(-*d_10_*); (13) anthranilic acid(-*^13^C_6_*); (14) indole-3-acetic acid(-*^13^C_6_*); (15) cortisol(-*^13^C_3_*); (16) cortisone(-*^13^C_3_*); (17) dehydroisoandrosterone sulfate(-*d_5_*); (18) 11-deoxycortisol(-*^13^C_3_*); (19) androsterone 3α-glucuronide(-*d_4_*); and (20) corticosterone(-*d_4_*). (*): isobaric metabolites.

The separation of the target metabolites represents an analytical challenge in terms of chemical diversity, having logD_pH=2.7_ ranging from −2.95 (3-hydroxykynurenine) to 3.36 (dehydroisoandrosterone sulfate), the presence of isobaric compounds requiring chromatographic separation, along with a broad range of concentrations to be quantified, ranging from 18 pg/mL (11-deoxycortisol) to 37 μg/mL (tryptophan). Formic acid was selected as an additive in mobile phases as most of the metabolites were acids or neutral compounds. On the contrary, a screening of stationary reverse phase columns was carried out including a variety of bonded ligands, such as trifunctional octadecyl, octa, propyl with cyano group, trifunctional octadecyl with charge modification, propylfluorophenyl and biphenyl. The chromatographic selectivity was focused on isobaric compounds that cannot be differentiated with the mass spectrometer (Figure S1). Also, an important parameter was the elution order of the analytes to obtain the best compromise in terms of mass spectrometer duty cycle. The biphenyl stationary phase provided an optimal chromatographic retention of semi-polar metabolites along with the baseline separation of isobaric compounds (Figure 2).

Additionally, electrospray interface ionization was greatly improved by including a post-column infusion of ammonium fluoride (Figure S2).^33^ As their specificity can be compromised in complex samples such as serum or plasma, transitions including water (18 *m/z*) or carbon dioxide losses (44 *m/z*) were excluded when possible. For each analyte and SIL, at least two MRM transitions were selected and their ratio monitored over the LC–MS/MS analysis to ensure method specificity. Also, the possibility to perform fast polarity switching with triple quadrupole instruments allowed an increased confidence on some analytes by monitoring positive and negative ionization, as it is the case for metabolites with short retention times such as *n*-acetyl-alanine (Table S7).

To perform the internal calibration (IC) approach, appropriate linear response functions need to be established before routine implementation. The high concentration range to be covered constituted a challenge regarding the linear dynamic range of the instrument. In addition, the elevated upper limit of quantification (ULOQ) required for the analysis of highly concentrated metabolites (>10 μg/mL, e.g., tryptophan) or the wide dynamic range (>2 magnitude order, such as kynurenic acid) resulted in a nonlinear response caused by ionization saturation. To overcome this, fast polarity switching was used, and the analytes in question were determined by negative ionization (Figure S3).

### Internal Calibration

The IC approach can be performed when a suitable surrogate calibrant with very similar physicochemical proprieties compared to the authentic analytes is available.^34^ Stable isotope-labeled (SIL) standards are the preferred choice because of their chemical similarity with unlabeled analyte, leading to comparable behaviors during the sample preparation and measurement process. Recently, the increased commercial accessibility of SIL analogues opened the possibility to perform MTIC quantification. In this work, their use as surrogate calibrants was carefully designed to mitigate any ionization competition and isotopic contribution to, or from, unlabeled metabolite analogues, or any other calibrator. SILs with high levels of chemical purity (>97%) and isotopic enrichment (>96%) were selected to mitigate the risk of interference between authentic analytes and SILs (Table S1). Moreover, semi-automated electronic pipetting was implemented in the workflow to reduce variability when preparing SIL mixtures batches.

The determination of SIL spiked concentration was a key step in method development to achieve accurate quantification based on internal calibration, as there are currently no guidelines for this emerging methodology. Published methods usually set SIL concentration at the upper dynamic range to perform interpolation, but this strategy can lead to an alteration of the linear concentration-response relationship.^23^ To determine the optimal SIL concentration and establish the behavior between the SIL and the corresponding analyte in LC–MS/MS, six calibration curves with specific concentration ranges for each analyte were spiked with different amounts of SILs, namely, 0, 12.5, 25, 50, 75, and 100% of the expected upper limit of biological concentration based on the published literature (Figure S4).^10^ The analyte response function obtained in the absence of the corresponding SIL was used as reference to compare the effect of different SIL concentrations via analysis of covariance of the slope and the intercept. The lowest SIL concentrations that did not alter the corresponding analyte MS response were selected (Table S4). For all metabolites, the effect of selected SIL analogue concentrations on the analyte response function was not significantly altering neither the slope nor the intercept (Table S8).

### Quantification Performance Evaluation

The quantification performance was achieved using a pooled human serum QC sample with reference values determined by the standard addition method, which is the highest metrological quantification methodology in LC–MS.^35^ The pooled QC was sequentially diluted with depleted serum to estimate the lower limit of quantification (LLOQ). For this reason, human plasma was discarded for performance evaluation because charcoal removes fibrinogens resulting in depleted serum.^36^ The limit of quantification was set for each metabolite at the lowest concentration that resulted in an interassay CV of <20% in QC serum samples.

The performance results, summarized in Table S9, showed that the MTIC method using a microsampling technique as a volumetric collection of human serum was precise, with repeatability and intermediate precision values ranging from 1.9 to 15.4% over four days of validation. Overall, the recovery values (trueness) ranged from 72.2 to 116%, with the exception of phenyl-acetylglutamine and *n*-acetyl-leucine which showed biases higher than 30%. Only 3-hydroxyanthranilic acid, 11-deoxycortisol, and phenylacetyl-glutamine showed inter-assay values higher than 20%, probably due to an increased RF variability between validation days (Table S8). *N*-Acetyl-leucine presented acceptable precision (3.5–11.0%) with a constant negative bias of 30%, suggesting that the matrix effect was inadequately corrected possibly due to high deuterium replacement in the SIL analogue. These results suggest that the performance of the LC–MS/MS method, together with the simplified volumetric collection and the calibration approach, was suitable to detect concentration differences between control and the chronic kidney disease progression (Table S10).

### Application to Clinical Study Samples

Serum creatinine and cystatin C are widely used as endogenous biomarkers of kidney function. Their role is however restricted physiologically to GFR estimation and clinically to CKD stratification, offering very limited insight into other aspects of kidney function and overall clinical guidance. Moreover, their concentration is confounded by various non-eGFR determinants frequently encountered in clinical practice such as muscle mass, diet, inflammation, smoking, medications, and hormonal status.^37^ In this work, the developed LC–MS/MS multi-targeted quantitative method was thus tested on an independent cohort of CKD patients to assess its potential clinical value.

**Figure 3:**
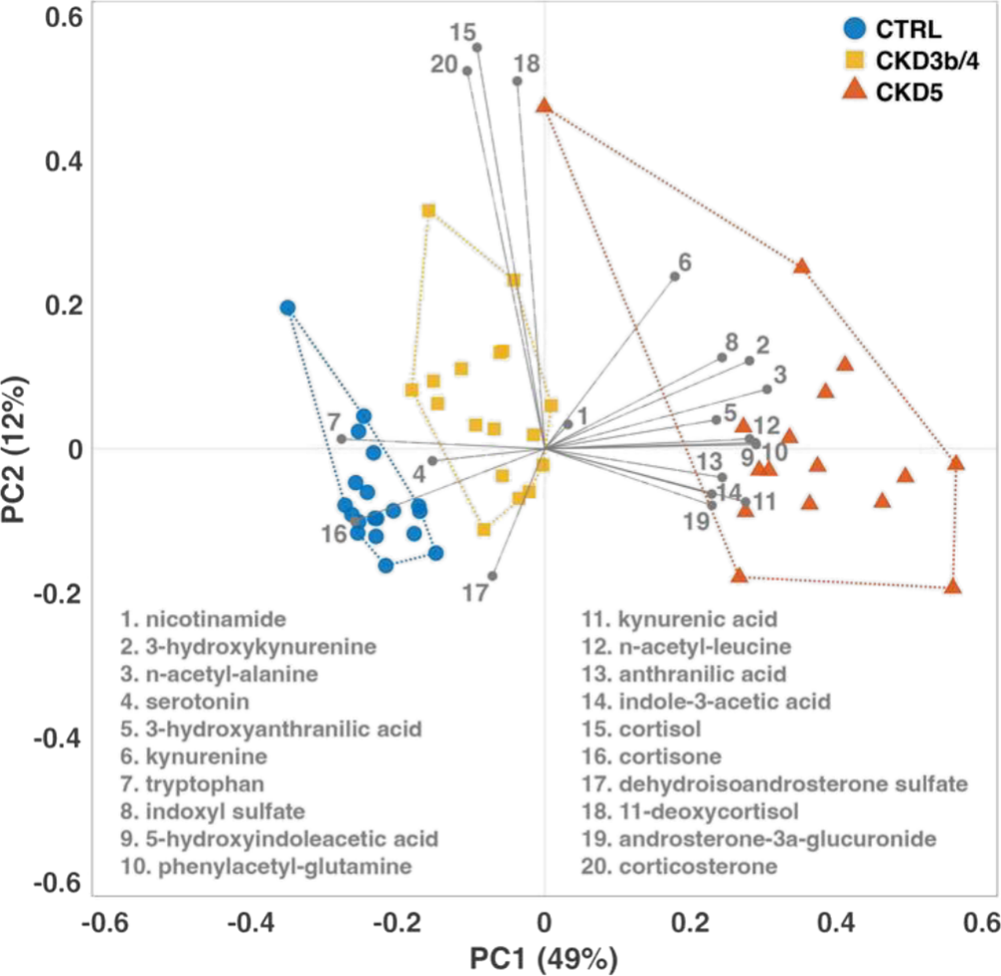
Biplot of principal component analysis of CTRL, CKD3b/4, and CKD5.

**Figure 4:**
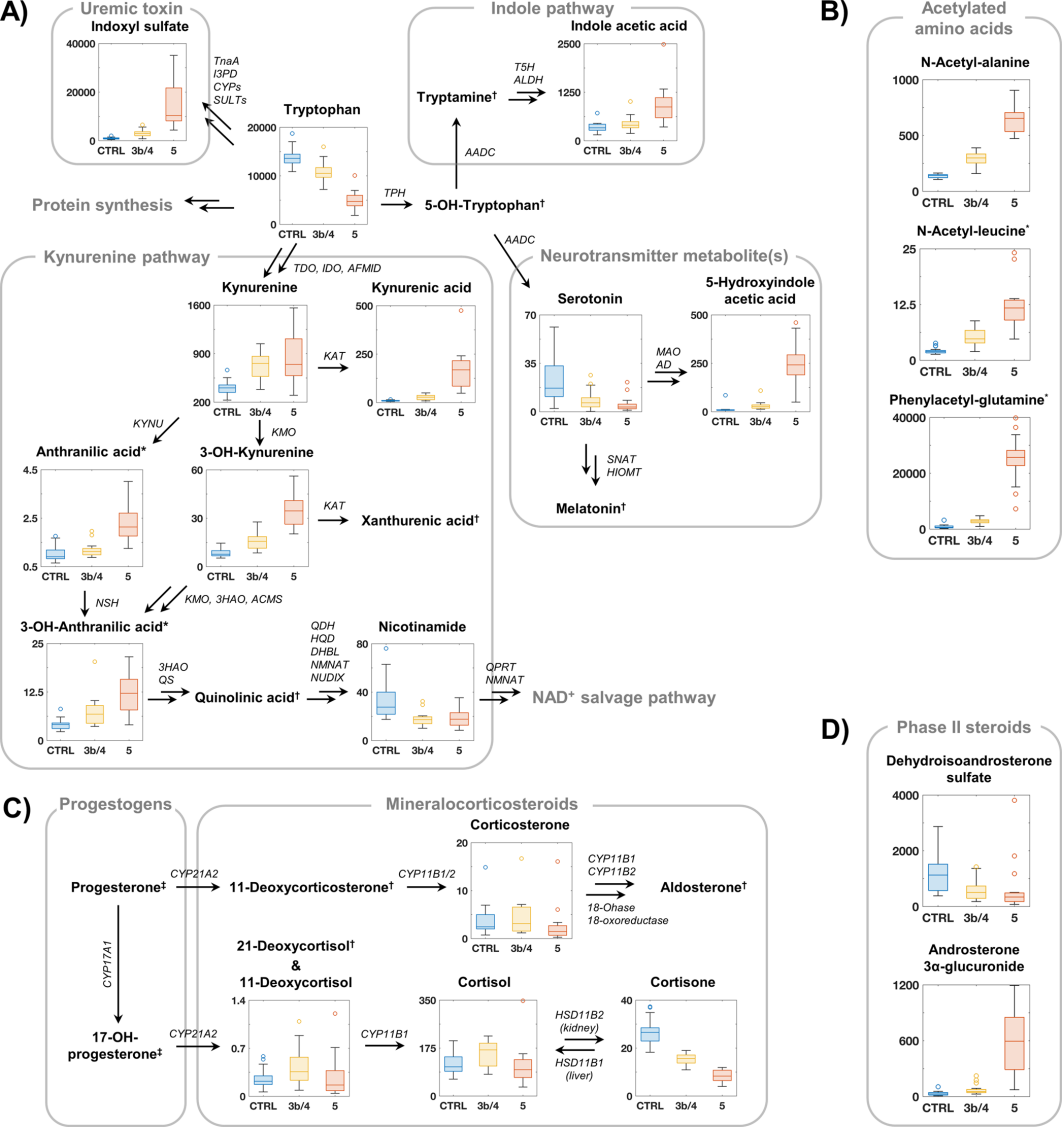
Box plots showing differences between control (CTRL) and the different stages of the CKD progression (3b/4 and 5) in (A) tryptophan pathway; (B) modified amino acids; (C) mineralocorticosteroids pathway; and (D) phase II steroids. All values are in ng/mL. (*): analytes partially validated; (†): metabolites monitored but not validated; (‡): compounds not included in the method. For enzyme abbreviation, see Table S11.

Principal component analysis (PCA) after unit variance scaling highlighted differences between the control group and CKD patients, as well as between the disease stages based on quantified patterns of metabolites. A significant trend in the metabolic profiles was observed according to the progression of the disease as the first axis represented 49% of the total variability (Figure 3). Overall, our platform was thus able to reliably characterize a wide spectrum of kidney dysfunction in a clinically relevant fashion, based on metabolic profiles alone. The quantification aspect of the proposed approach increases the confidence in the monitored metabolites as well as the possibility to obtain absolute concentration values to monitor CKD disease progression. Metabolite contributions were then investigated by plotting the box plots of each monitored analyte onto the corresponding biological pathway (Figure 4). We observed a decrease in tryptophan levels across CKD stages. On the opposite, kynurenine increased with CKD severity. In agreement with previous findings, this pattern likely reflects increase indoleamine 2,3-dioxygenase (IDO) activity in the setting of kidney disease (Figure 4A).^38^ Monitoring multiple kynurenine metabolites allowed CKD3b/4 and CKD5 subgroups to be distinguished, with markedly elevated kynurenic acid and 3-hydrokynurenine concentrations for the latter, reflecting increased kynurenine aminotransferase (KAT) activity.^39^ Of note, indoxyl sulfate, a well-described protein-bound uremic toxin, was also markedly elevated in late CKD stages, in line with clinical evidences.^40^ Also, disease progression seems to be correlated with a decrease in cortisone concentration, suggesting a reduction of the enzyme 11β-hydroxysteroid dehydrogenase (HSD11B2) associated to the interconversion between cortisol and cortisone in the kidney (Figure 4C).^41^ In addition to the tryptophan and mineralocorticosteroids pathways, some acetylated amino acids and some phase II steroids were also monitored because they are waste products, and their accumulation may be associated with chronic kidney disease due to impaired kidney function (Figure 4B,D).^42^

## Conclusions

To the best of our knowledge, this is the first published targeted method for monitoring and quantifying multiple biological pathways simultaneously using a calibration performed directly in study samples. The use of a single internal calibrator eliminates the need for a blank matrix and the extensive preparation of multilevel calibration curves, as well as the need for matrix effect studies because calibrants are not spiked into a separate matrix. However, the use of internal calibration should be carefully evaluated to minimize any cross contribution or ionization competition, especially when a wide range of concentrations (>100 fold) needs to be covered.

Moreover, the results demonstrated that internal calibration of endogenous metabolite quantification is expected to improve throughput and random analysis by eliminating the need for batch preparation. Because the microsampling strategy does not require a specialized operator and the biological sample can be collected directly from the sampling tube, the proposed MTIC method can easily be implemented into clinical routine. Finally, the simplicity of sample preparation and LC–MS quantification makes it suitable for implementation in clinical laboratories. Future research will focus on applying this method for longitudinal monitoring of relevant biomarkers in CKD patients using individualized and adaptive reference ranges.

## Supporting Information Available

Chromatographic separation of isobaric mineralocorticosteroids; signal enhancement factor using ammonium fluoride as a postcolumn additive; transition de-optimization to ensure instrument response linearity; SIL concentration effect on corresponding analyte instrument function (visualization and statistical output); chemical purity, isotopic enrichment, and solvent used for all chemicals used; labeled and unlabeled standard mix concentrations used for instrument response function evaluation; selected concentrations of SIL to perform the internal calibration; human plasma and serum QC target concentrations for method performance assessment; mass spectrometric conditions and chromatographic retention time; trueness and precision of the internal calibration approach; baseline healthy volunteers and CKD patient characteristics; and enzyme abbreviation list (PDF)
