# Supplementary material for: Multitargeted Internal Calibration for the Quantification of Chronic Kidney Disease-Related Endogenous Metabolites Using Liquid Chromatography–Mass Spectrometry
Source: Anal Chem. 2023 Sep 1;95(36):13546–54. doi: 10.1021/acs.analchem.3c02069 (PMC10500547; doi:10.1021/acs.analchem.3c02069)
Supplement: Supplementary file 1 — ac3c02069_si_001.pdf [file ac3c02069_si_001.pdf]

SUPPORTING INFORMATION

Multitargeted internal calibration for the quantification of chronic kidney disease related endogenous metabolites using liquid chromatography–mass spectrometry

AUTHORS: Gioele Visconti[1,2], Miguel de Figueiredo[1,2], Oriane Strassel[1,2], Julien Boccard[1,2], Nicolas Vuilleumier[3], David Jaques[4], Belén Ponte[4], Serge Rudaz[1,2*]

(1) School of Pharmaceutical Sciences, University of Geneva, 1211 Geneva 4, Switzerland;
(2) Institute of Pharmaceutical Sciences of Western Switzerland, University of Geneva, 1211 Geneva 4, Switzerland;
(3) Department of Genetic and Laboratory Medicine, Geneva University Hospitals (HUG), 1205 Geneva, Switzerland;
(4) Service of Nephrology, Geneva University Hospitals (HUG), 1205 Geneva, Switzerland;

*corresponding author**:** serge.rudaz@unige.ch (Pr. Serge Rudaz)

Figure S1: Chromatographic separation of isobaric mineralocorticosteroids S2

Figure S2: Signal enhancement factor using NH_4F as a postcolumn additive S3

Figure S3: Transitions de-optimization to ensure instrument response linearity S4

Figure S4: SIL concentration effect on corresponding analyte instrument function S5

Table S1: Chemical purity, isotopic enrichment and solvent used for all chemical used S6

Table S2: Unlabeled standard mixes for instrument response function evaluation S8

Table S3: Labeled standard mixes for instrument response function evaluation S9

Table S4: Selected concentrations of SIL to perform the internal calibration S10

Table S5: Human plasma QCs target concentrations S11

Table S6: Human serum QCs target concentrations S12

Table S7: Mass spectrometric conditions and chromatographic retention time S13

Table S8: Response linearity and response factor stability evaluation S14

Table S9: Trueness and precision of the internal calibration approach S15

Table S10: Baseline healthy volunteers and CKD patient characteristics S16

Table S11: Enzyme abbreviation list used in Figure 4 S17

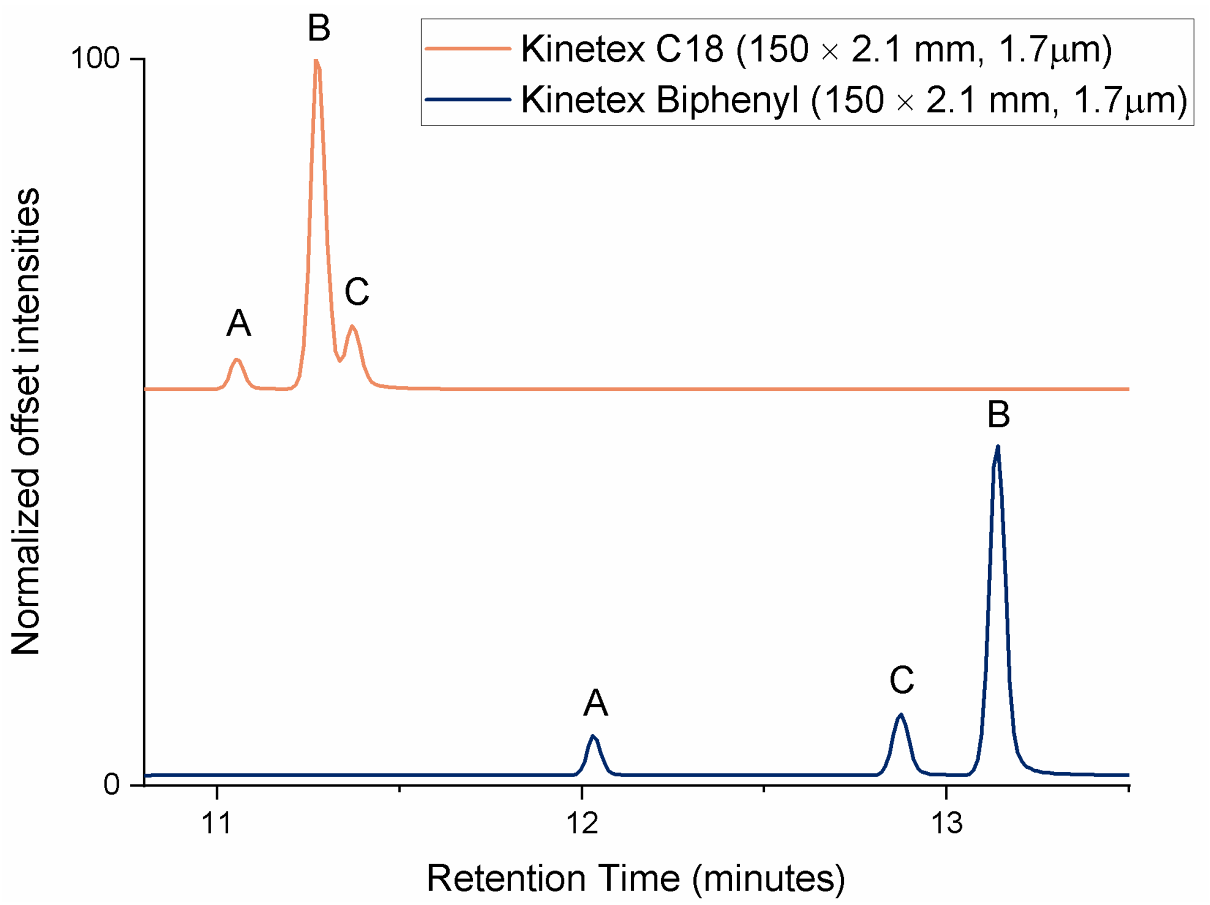

Figure S1. Chromatographic separation optimization of mineralocorticosteroids isomers: A) 21-deoxycortisol, B) corticosterone, and C) 11-deoxycortisol. The mobile phase conditions are the same as described in the principal manuscript.

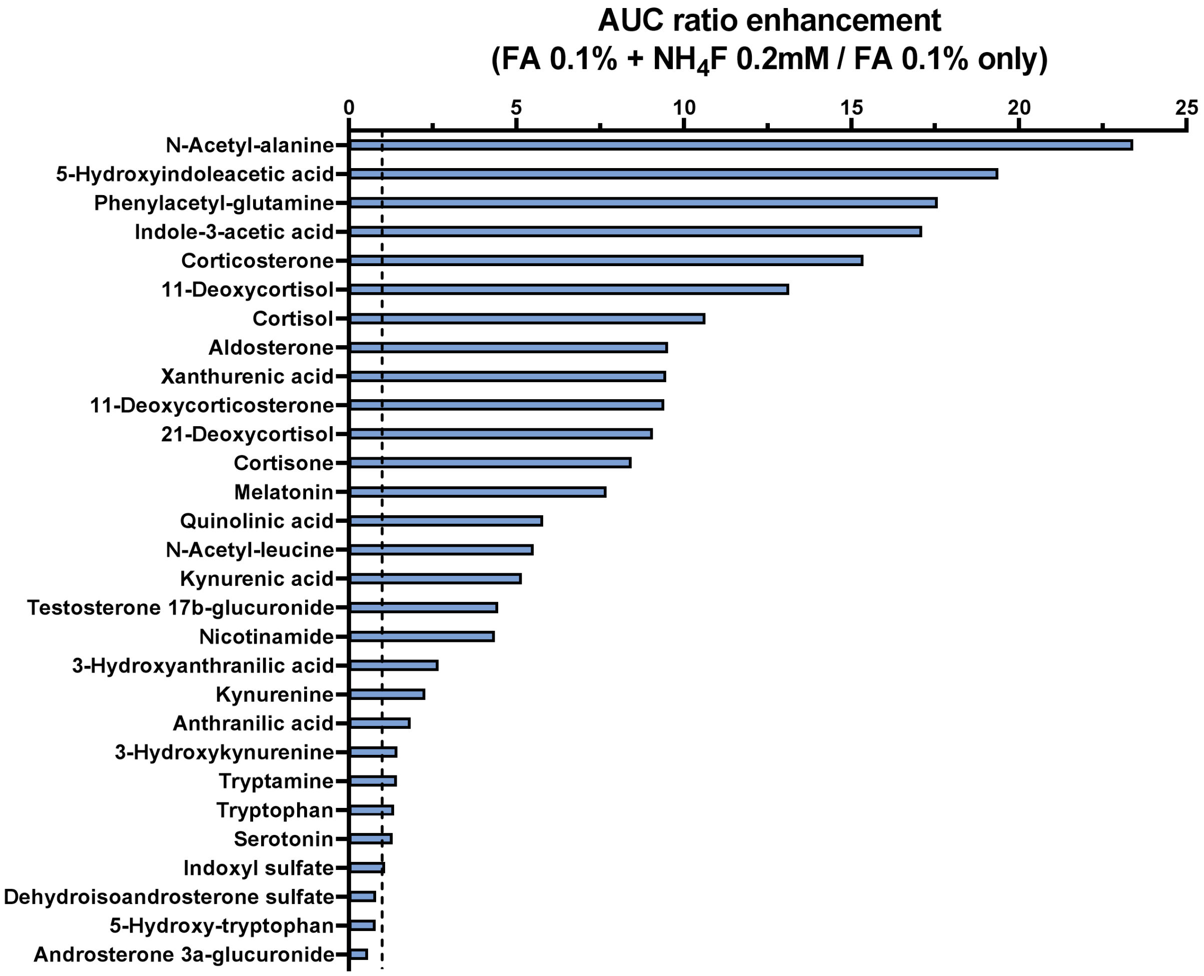

Figure S2. Signal enhancement using the post-column infusion of ammonium fluoride (NH_4F) at 0.2mM instead of formic acid (FA) 0.1% only. Dotted line represents the unit values, i.e., no changes in the ionization enhancement. AUC: area under the curve.

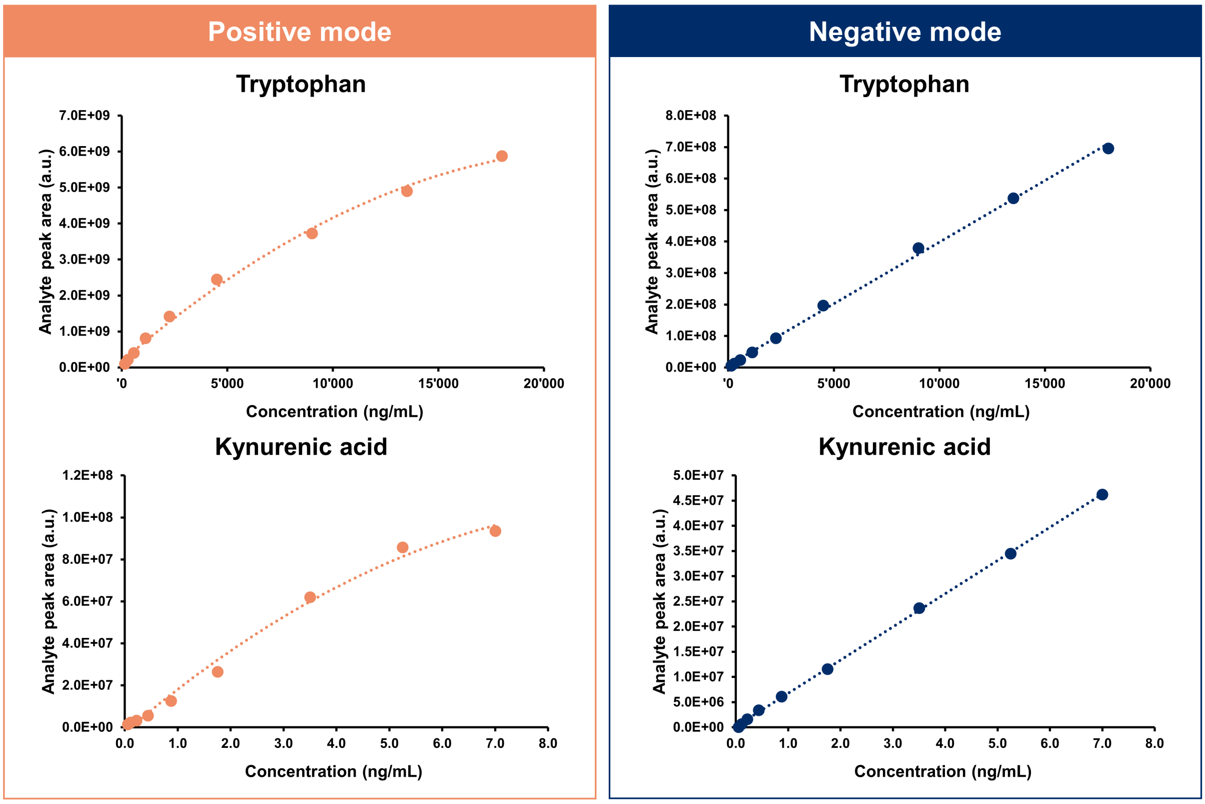

Figure S3. Transitions de-optimization to avoid detection saturation on the explored dynamic range (Table S2).

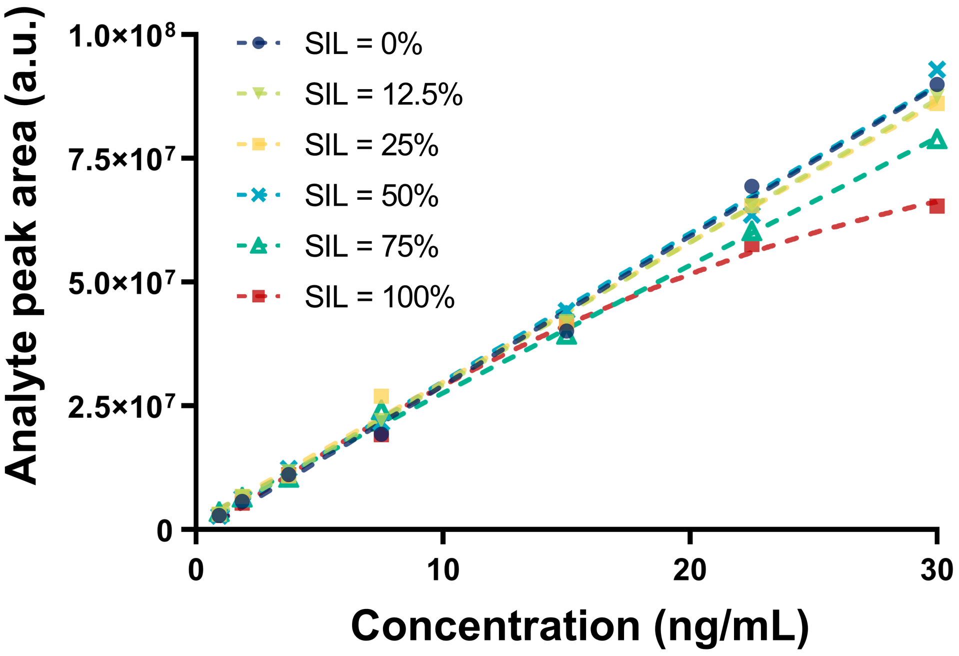

Figure S4. SIL concentration effect on analyte response function, using the 3-hydroxyanthranilic acid and its labeled analog (3-hydroxyanthranilic acid-d_3) as example. The analyte response function obtained in absence of the corresponding SIL was used as reference to compare the effect of different SIL concentrations via analysis of covariance (Table S7).

Table S1: Chemical purity, isotopic enrichment and solvent used for the analytes and their corresponding stable isotope-labeled analogs. N/A: not applicable. DMSO: dimethyl sulfoxide, H_2O: water, MeOH: methanol, NaOH: sodium hydroxide, FA: formic acid.

Compound	Chemical purity (%)	Isotopic enrichment (%)	Solvent
3-Hydroxy anthranilic acid	100.0	N/A	DMSO
3-Hydroxy anthranilic acid-d_3	97.0	96.0	
5-Hydroxyindoleacetic acid	98.5	N/A	H_2O/MeOH 50:50 + 10mM ascorbic acid
5-Hydroxyindoleacetic acid-$^{13}C_6$	100.0	99.5	
Anthranilic acid	100.0	N/A	
Anthranilic acid-$^{13}C_6$	98.0	99.8	
Indole-3-acetic acid	98.4	N/A	
Indole-3-acetic acid-$^{13}C_6$	95.0	99.0	
Indoxyl sulfate	99.7	N/A	
Indoxyl sulfate-$^{13}C_6$	99.4	99.0	
Kynurenine	99.0	N/A	
Kynurenine-$^{13}C_6$	95.0	99.0	
N-acetyl-alanine	98.0	N/A	
N-acetyl-alanine-d_3	96.0	99.9	
N-acetyl-leucine	99.8	N/A	
N-acetyl-leucine-d_{10}	99.7	98.5	
Phenylacetyl-glutamine	99.2	N/A	
Phenylacetyl-glutamine-d_5	98.9	98.7	
Serotonin	98.5	N/A	
Serotonin-d_4	99.4	98.0	
Tryptophan	99.8	N/A	
Tryptophan-$^{13}C_{11}$	98.0	99.1	
Kynurenic acid	99.8	N/A	H_2O/MeOH 50:50 + 0.05M NaOH + 10mM ascorbic acid
Kynurenic acid-d_5	98.4	98.9	
3-Hydroxykynurenine	97.5	N/A	H_2O/MeOH 50:50 + 0.1M FA + 10mM ascorbic acid
3-Hydroxykynurenine-$^{13}C_3$,^{15}N	98.0	99.6	
11-Deoxycortisol	99.0	N/A	MeOH
11-Deoxycortisol-$^{13}C_3$	99.0	99.0	
Androsterone 3α-glucuronide	87.2	N/A	
Androsterone 3α-glucuronide-d_4	97.3	92.9	
Corticosterone	98.6	N/A	
Corticosterone-d_4	99.0	99.0	
Cortisol	99.0	N/A	
Cortisol-$^{13}C_3$	98.0	99.0	
Cortisone	98.0	N/A	

Compound	Chemical purity (%)	Isotopic enrichment (%)	Solvent
Cortisone-$^{13}C_3$	97.0	100.0	
Dehydroisoandrosterone sulfate	99.9	N/A	
Dehydroisoandrosterone sulfate-d_5	99.8	99.3	
Nicotinamide	99.9	N/A	
Nicotinamide-$^{13}C_6$	99.8	99.4	

Table S2. Standard mix composition and final concentration in water for the instrument response linearity evaluation (ordered alphabetically). All values are in ng̃mL.

Compound	Cal 1	Cal 2	Cal 3	Cal 4	Cal 5	Cal 6	Cal 7	Cal 8	Cal 9
11-Deoxycortisol	0.01	0.01	0.03	0.05	0.10	0.20	0.40	0.60	0.80
3-Hydroxyanthranilic acid	0.23	0.47	0.94	1.88	3.75	7.50	15.00	22.50	30.00
3-Hydroxykynurenine	1.80	3.59	7.19	14.38	28.75	57.50	115.00	172.50	230.00
5-Hydroxyindoleacetic Acid	0.09	0.19	0.38	0.75	1.50	3.00	6.00	9.00	12.00
Androsterone 3a-glucuronide	0.31	0.63	1.25	2.50	5.00	10.00	20.00	30.00	40.00
Anthranilic acid	0.0016	0.0031	0.0063	0.0125	0.0250	0.0500	0.1000	0.1500	0.2000
Corticosterone	0.12	0.23	0.47	0.94	1.88	3.75	7.50	11.25	15.00
Cortisol	2.34	4.69	9.38	18.75	37.50	75.00	150.00	225.00	300.00
Cortisone	0.31	0.63	1.25	2.50	5.00	10.00	20.00	30.00	40.00
Dehydroisoandrosterone sulfate	27.34	54.69	109.38	218.75	437.50	875.00	1 750.00	2 625.00	3 500.00
Indole-3-acetic acid	6.64	13.28	26.56	53.13	106.25	212.50	425.00	637.50	850.00
Indoxyl sulfate	6.64	13.28	26.56	53.13	106.25	212.50	425.00	637.50	850.00
Kynurenic Acid	0.05	0.11	0.22	0.44	0.88	1.75	3.50	5.25	7.00
Kynurenine	3.13	6.25	12.50	25.00	50.00	100.00	200.00	300.00	400.00
N-acetyl-alanine	4.69	9.38	18.75	37.50	75.00	150.00	300.00	450.00	600.00
N-acetyl-leucine	4.69	9.38	18.75	37.50	75.00	150.00	300.00	450.00	600.00
Nicotinamide	0.04	0.08	0.16	0.31	0.63	1.25	2.50	3.75	5.00
Phenylacetyl-glutamine	31.25	62.50	125.00	250.00	500.00	1 000.00	2 000.00	3 000.00	4 000.00
Serotonin	1.37	2.73	5.47	10.94	21.88	43.75	87.50	131.25	175.00
Tryptophan	140.63	281.25	562.50	1 125.00	2 250.00	4 500.00	9 000.00	13 500.00	18 000.00

Table S3. Stable isotope-labeled (SIL) mix composition and concentrations for the instrument response linearity evaluation (ordered alphabetically). All values are in ng̃mL.

Compound	SIL 5	SIL 6	SIL 7	SIL 8	SIL 9
11-Deoxycortisol-$^{13}C_3$	0.1003	0.2005	0.4010	0.6015	0.8000
3-Hydroxyanthranilic acid-d_3	3.76	7.52	15.04	22.56	30.00
3-Hydroxykynurenine-$^{13}C_3$, ^{15}N	28.82	57.64	115.29	172.93	230.00
5-Hydroxyindoleacetic acid-$^{13}C_6$	1.50	3.01	6.02	9.02	12.00
Androsterone glucuronide-d_4	5.01	10.03	20.05	30.08	40.00
Anthranilic acid-$^{13}C_6$	0.0251	0.0501	0.1003	0.1504	0.2000
Corticosterone-d_4	1.88	3.76	7.52	11.28	15.00
Cortisol-$^{13}C_3$	37.59	75.19	150.38	225.56	300.00
Cortisone-$^{13}C_3$	5.01	10.03	20.05	30.08	40.00
Dehydroisoandrosterone sulfate-d_5	219.85	439.70	879.39	1 319.09	1 754.39
Indole-3-acetic acid-$^{13}C_6$	106.52	213.03	426.07	639.10	850.00
Indoxyl sulfate-$^{13}C_6$	106.52	213.03	426.07	639.10	850.00
Kynurenic acid-d_5	0.88	1.75	3.51	5.26	7.00
Kynurenine-$^{13}C_6$	50.13	100.25	200.50	300.75	400.00
N-Acetyl-alanine-d_3	75.19	150.38	300.75	451.13	600.00
N-Acetyl-leucine-d_{10}	75.19	150.38	300.75	451.13	600.00
Nicotinamide-$^{13}C_6$	0.63	1.25	2.50	3.75	5.00
Phenylacetyl glutamine-d_5	501.25	1 002.51	2 005.01	3 007.52	4 000.00
Serotonin-d_4	21.93	43.86	87.72	131.58	175.00
Tryptophan-$^{13}C_{11}$	2 250.00	4 500.00	9 000.00	13 500.00	18 000.00

Table S4. Stable isotope-labeled mix (SIL-mix) composition and final concentrations for the quantification in biological samples (ordered alphabetically).

Compound	Concentration in ng/mL
11-Deoxycortisol-$^{13}C_3$	0.02
3-Hydroxy anthranilic acid-d_3	1.50
3-Hydroxykynurenine-$^{13}C_3$, ^{15}N	1.00
5-Hydroxyindoleacetic acid-$^{13}C_6$	0.90
Androsterone glucuronide-d_4	3.00
Anthranilic acid-$^{13}C_6$	0.01
Corticosterone-d_4	0.20
Cortisol-$^{13}C_3$	3.75
Cortisone-$^{13}C_3$	1.00
Dehydroisoandrosterone sulfate-d_5	87.50
Indole-3-acetic acid-$^{13}C_6$	21.25
Indoxyl sulfate-$^{13}C_6$	10.63
Kynurenic acid-d_5	0.20
Kynurenine-$^{13}C_6$	40.00
N-acetyl-alanine-d_3	30.00
N-acetyl-leucine-d_{10}	0.75
Nicotinamide-$^{13}C_6$	3.00
Phenylacetyl glutamine-d_5	50.00
Serotonin-d_4	2.19
Tryptophan-$^{13}C_{11}$	450.00

Table S5. Human plasma QCs target concentrations values (in ng/mL, ordered alphabetically).

Analyte	Endo	Mid	High
11-Deoxycortisol	0.10	0.51	0.93
3-Hydroxy anthranilic Acid	2.71	12.20	21.68
3-Hydroxykynurenine	5.83	10.59	15.35
5-Hydroxyindoleacetic Acid	6.34	17.51	28.67
Androsterone 3a-glucuronide	16.96	39.57	62.19
Anthranilic acid	0.68	1.14	1.61
Corticosterone	1.40	7.88	14.35
Cortisol	64.02	206.98	349.95
Cortisone	12.05	31.44	50.83
DHEAS	540.76	1’565.95	2’591.14
Indole-3-Acetic Acid	206.04	792.49	1’378.94
Indoxyl Sulfate	496.51	1’451.53	2’406.54
Kynurenic Acid	6.06	13.46	20.86
Kynurenine	303.56	591.87	880.19
N-Acetyl-L-Alanine	103.82	407.43	711.04
N-Acetyl-L-Leucine	1.70	21.00	40.31
Nicotinamide	21.52	79.16	136.81
Phenylacetyl-glutamine	285.53	1’999.52	3’713.50
Serotonin	11.24	85.71	160.17
Tryptophan	8’548.71	21’214.67	33’880.63

Table S6. Human serum QCs target concentrations values (in ng/mL, ordered alphabetically). LLOQ: lower limit of quantification, LOQ: limit of quantification, Endo: pooled endogenous concentrations determined by the standard addition method, ULOQ: upper limit of quantification. “LLOQ”, “LOQ” and “Low” are dilutions of “Endo” with depleted serum. “Mid” and “ULOQ” are spiked “Endo” samples.

Analyte	LLOQ	Low 1	Low 2	Endo	Mid	ULOQ
11-Deoxycortisol	0.02	0.04	0.08	0.13	0.54	0.96
3-Hydroxyanthranilic acid	0.33	0.98	1.96	3.27	12.76	22.24
3-Hydroxykynurenine	0.60	1.80	3.61	6.01	10.77	15.53
5-Hydroxyindoleacetic acid	1.46	4.39	8.78	14.63	25.79	36.96
Androsterone 3α-glucuronide	2.47	7.41	14.83	24.71	47.33	69.94
Anthranilic acid	0.10	0.29	0.58	0.97	1.44	1.90
Corticosterone	0.14	0.43	0.85	1.42	7.90	14.37
Cortisol	7.33	22.00	44.00	73.34	216.31	359.27
Cortisone	1.62	4.86	9.73	16.21	35.60	54.99
Dehydroisoandrosterone sulfate	146.78	440.33	880.67	1’467.78	2’492.97	3’518.15
Indole-3-acetic acid	30.22	90.66	181.32	302.19	888.65	1’475.10
Indoxyl sulfate	119.13	357.40	714.79	1’191.32	2’146.33	3’101.35
Kynurenic acid	0.67	2.02	4.05	6.75	14.15	21.56
Kynurenine	37.63	112.89	225.78	376.30	664.61	952.93
N-acetyl-alanine	14.73	44.20	88.41	147.34	450.95	754.56
N-acetyl-leucine	0.37	1.12	2.24	3.73	23.03	42.34
Nicotinamide	3.32	9.95	19.89	33.16	90.80	148.45
Phenylacetyl-glutamine	33.70	101.09	202.18	336.97	2’050.96	3’764.95
Serotonin	10.00	30.01	60.02	100.03	174.50	248.97
Tryptophan	1’197.13	3’591.39	7’182.78	11’971.30	24’637.26	37’303.22

Table S7. Mass spectrometric conditions and chromatographic retention time for each metabolite and stable-labeled isotopic standard (ordered by retention time).

RT (min)	Metabolite name	Polarity	Precursor ion (m/z)	Product ion (m/z) QT; QL	Collision energy (eV)	Cone voltage (V)
2.16	Nicotinamide	Pos	123.1	80.0 ; 106.0	16 ; 14	28
	Nicotinamide-$^{13}C_6$	Pos	129.1	85.0 ; 112.0	16 ; 14	28
2.38	3-Hydroxykynurenine	Pos	225.2	110.0 ; 162.0	23 ; 23	23
	3-Hydroxykynurenine-$^{13}C_3$,^{15}N	Pos	229.2	110.0 ; 164.0	23 ; 23	23
2.43	N-acetyl-alanine-d_3	Pos / Neg*	133.1* ; 135.1	90.9 ; 92.9	14 ; 8	23 ; 14
2.44	N-acetyl-alanine	Pos / Neg*	130.1* ; 132.1	87.9 ; 89.9	14 ; 8	23 ; 14
3.48	Serotonin-d_4	Pos	181.1	164.0 ; 136.0	14 ; 23	18
3.50	Serotonin	Pos	177.1	160.0 ; 132.0	14 ; 23	18
4.15	3-Hydroxyanthranilic acid-d_3	Pos* / Neg	140.1*,§ ; 155.1	111.0 ; 111.0	12 ; 12	20 ; 28
4.18	3-Hydroxyanthranilic acid	Pos* / Neg	136.1*,§ ; 152.1	108.0 ; 108.0	12 ; 12	20 ; 28
4.87	Kynurenine	Pos	209.1	94.0 ; 146.0	14 ; 23	23
	Kynurenine-$^{13}C_6$	Pos	215.1	100.0 ; 152.0	14 ; 23	23
5.10	Tryptophan	Neg	203.2	115.9 ; 73.9	23 ; 23	23
	Tryptophan-$^{13}C_{11}$	Neg	214.2	123.9 ; 75.9	23 ; 23	23
5.20	Indoxyl sulfate	Neg	212.0	131.9 ; 79.9	23 ; 23	32
	Indoxyl sulfate-$^{13}C_6$	Neg	218.0	137.9 ; 79.9	23 ; 23	32
5.83	5-Hydroxyindoleacetic acid	Pos	192.1	146.0 ; 91.0	14 ; 32	23
	5-Hydroxyindoleacetic acid-$^{13}C_6$	Pos	198.1	152.0 ; 96.0	14 ; 33	24
6.59	Phenylacetyl-glutamine-d_5	Neg	268.1	144.9 ; 126.9	16 ; 32	6
6.65	Phenylacetyl-glutamine	Neg	263.1	144.9 ; 126.9	16 ; 32	6
6.88	Kynurenic acid-d_5	Pos / Neg*	193.1* ; 195.1	149.0 ; 121.0	14 ; 32	5 ; 8
6.89	Kynurenic acid	Pos / Neg*	188.1* ; 190.1	144.0 ; 116.0	14 ; 32	5 ; 8
6.97	N-acetyl-leucine-d_{10}	Pos / Neg*	182.1* ; 184.1	140.0 ; 96.0	14 ; 16	5 ; 3
7.07	N-acetyl-leucine	Pos / Neg*	172.1* ; 174.1	130.0 ; 86.0	14 ; 16	5 ; 3
7.15	Anthranilic acid	Pos	138.1* ; 120.1§	120.1* ; 92.0	14 ; 14	14 ; 42
	Anthranilic acid-$^{13}C_6$	Pos	144.1* ; 126.1§	126.1* ; 98.0	14 ; 14	14 ; 42
8.47	Indole-3-acetic acid	Pos	176.1	103.0 ; 77.0	28 ; 41	4
	Indole-3-acetic acid-$^{13}C_6$	Pos	182.1	109.0 ; 81.0	28 ; 41	4
11.57	Cortisol	Pos	363.2	97.0 ; 121.1	32 ; 24	24
	Cortisol-$^{13}C_3$	Pos	366.2	100.1 ; 124.1	32 ; 24	24
11.95	Cortisone	Pos	361.2	163.0 ; 121.1	24 ; 32	32
	Cortisone-$^{13}C_3$	Pos	364.2	166.1 ; 124.1	24 ; 32	32
12.78	Dehydroisoandrosterone sulfate-d_5	Neg	372.2	98.1 ; 79.9	41 ; 66	68
12.80	Dehydroisoandrosterone sulfate	Neg	367.2	97.1 ; 79.9	41 ; 66	68
13.09	11-Deoxycortisol	Pos	347.2	97.1 ; 109.1	32 ; 28	32
	11-Deoxycortisol-$^{13}C_3$	Pos	350.2	100.1 ; 112.1	32 ; 28	32
13.36	Androsterone 3α-glucuronide-d_4	Neg	469.2	74.9 ; 84.9	41 ; 34	78
13.37	Androsterone 3α-glucuronide	Neg	465.2	74.9 ; 84.9	41 ; 34	78
13.39	Corticosterone-d_4	Pos	351.2	121.1 ; 333.1	26 ; 11	38
13.41	Corticosterone	Pos	347.2	121.1 ; 97.1	26 ; 32	38

(Pos): positive ionization, (Neg): negative ionization, (Pos / Neg): polarity switching, *: selected transition when polarity switching is used, §: precursor with loss of water, QT: quantifier, QL: qualifier.

Table S8. SIL concentration effect on instrument response linearity and response factor (RF) stability evaluation (ordered alphabetically). The analysis of covariance comparison is performed by comparing the analyte instrumental response without the presence of SIL. (*): concentration was set to the closest analyte class as no reference interval was available.

Analyte	SIL analogue	Biological reference interval (ng/mL)	SIL concentration (ng/mL)	SIL concentration (% upper range)	R^2	Slope similarity (P value)	Intercept similarity (P value)	RF ± STD
11-Deoxycortisol	$-^{13}C_3$	0.00 - 0.76	0.2	25%	0.991	0.30	0.71	1.39 ± 0.07
3-Hydroxyanthranilic acid	$-d_3$	1.00 - 28.0	15.0	50%	0.989	0.50	0.36	0.91 ± 0.12
3-Hydroxykynurenine	$-^{13}C_3,^{15}N$	0.00 - 228	10.0	13%	1.000	0.44	0.82	1.35 ± 0.06
5-Hydroxyindoleacetic acid	$-^{13}C_6$	8.60 - 11.2	9.0	75%	0.999	0.43	0.05	0.85 ± 0.04
Androsterone 3α-glucuronide	$-d_4$	11.0 - 40.0	30.0	25%	0.988	0.55	0.59	0.92 ± 0.10
Anthranilic acid	$-^{13}C_6$	0.13 - 0.20	0.1	50%	0.980	0.51	0.44	1.02 ± 0.01
Corticosterone	$-d_4$	0.29 - 13.3	2.0	75%	0.999	0.30	1.00	1.00 ± 0.10
Cortisol	$-^{13}C_3$	23.5 - 252.5	37.5	13%	0.987	0.60	0.42	1.09 ± 0.03
Cortisone	$-^{13}C_3$	10.1 - 32.6	10.0	25%	0.992	0.45	0.82	1.04 ± 0.05
Dehydroisoandrosterone sulfate	$-d_5$	337 - 3'460	875.0	50%	0.994	0.65	0.27	1.46 ± 0.15
Indole-3-acetic acid	$-^{13}C_6$	200 - 800	212.5	25%	0.987	0.84	0.07	1.21 ± 0.01
Indoxyl sulfate	$-^{13}C_6$	240 - 820	106.3	13%	0.979	0.95	0.34	1.17 ± 0.03
Kynurenic acid	$-d_5$	4.40 - 7.00	2.0	13%	1.000	0.29	0.02	0.96 ± 0.05
Kynurenine	$-^{13}C_6$	320 - 400	400.0	100%	0.999	0.86	0.86	1.31 ± 0.01
N-acetyl-alanine	$-d_3$	230 - 570	300.0	50%	0.989	0.64	0.57	1.06 ± 0.07
N-acetyl-leucine	$-d_{10}$	230 - 570*	7.5	13%	1.000	0.64	0.79	1.1 ± 0.02
Nicotinamide	$-^{13}C_6$	2.4 - 4.9	30.0	13%	0.997	0.22	0.30	1.02 ± 0.05
Phenylacetyl-glutamine	$-d_5$	300 - 4'000	500.0	50%	0.989	0.88	0.93	0.71 ± 0.07
Serotonin	$-d_4$	136 - 163	21.9	13%	0.987	0.87	0.02	1.74 ± 0.07
Tryptophan	$-^{13}C_{11}$	6'000 - 17'500	4500.0	25%	0.986	0.90	0.60	0.78 ± 0.03

Table S9. Trueness and precision of the internal calibration approach (ordered alphabetically). LLOQ: lower limit of quantification, LOQ: limit of quantification, Endo: endogenous concentrations determined by the standard addition method, ULOQ: upper limit of quantification. For target concentrations details see Table S6. Values outside the acceptance limits are indicated with (*).

			Repeatability (CV%)						Intermediate Precision (CV%)						Trueness (%)					
Compound	LLOQ (ng/mL)	ULOQ (ng/mL)	LLOQ	LOQ	Low	Endo	Mid	ULOQ	LLOQ	LOQ	Low	Endo	Mid	ULOQ	LLOQ	LOQ	Low	Endo	Mid	ULOQ
11-Deoxycortisol	0.02	0.96	24.4*	11.3	18.2	4.2	8.5	5.5	28.7*	13.8	22.1*	10.9	12.6	11.3	109.4	115.7	115.2	105.5	104.4	105.1
3-Hydroxyanthranilic acid	0.40	22.32	15.8	7.4	3.9	4.1	8.1	3.1	28.4*	18.3	15.6	19.9	24.5*	19.8	68.3*	73.0	76.0	81.5	80.5	78.8
3-Hydroxykynurenine	0.67	15.6	5.8	5.2	6.4	3.9	4.2	4.6	27.1*	6.5	9.7	6.5	8.7	8.8	84.5	96.9	97.2	89.7	85.3	81.6
5-Hydroxyindoleacetic acid	1.47	36.96	5.1	5.2	4.5	3.1	3.5	3.4	6.2	5.6	6.0	3.2	4.6	3.5	92.5	94.6	92.8	88.7	88.0	87.0
Androsterone 3α-glucuronide	2.48	69.95	10.4	9.0	6.8	9.3	5.2	6.9	10.4	9.8	12.4	11.3	7.9	8.1	116.0	113.3	110.5	102.5	87.5	84.0
Anthranilic acid	0.31	2.11	4.1	5.2	3.8	4.4	4.2	3.6	16.7	10.0	7.4	6.6	6.6	5.1	69.1*	72.3	72.5	70.7	68.4*	67.9*
Corticosterone	0.15	14.37	9.4	5.5	5.0	5.4	3.5	3.3	18.4	11.6	13.9	15.4	13.9	13.4	109.5	108.2	107.1	103.8	91.0	90.9
Cortisol	7.39	359.3	4.0	5.5	3.5	2.4	4.2	3.9	4.4	5.7	3.8	3.4	4.2	4.2	108.7	109.1	109.0	105.4	102.3	101.3
Cortisone	1.63	55.0	4.5	5.5	2.7	2.6	4.4	3.7	6.3	6.2	5.7	3.0	5.0	3.7	104.0	104.7	103.2	99.3	96.5	96.9
Dehydroisoandrosterone sulfate	148.8	3’520.1	5.4	5.3	7.0	3.0	3.4	5.1	10.0	8.1	11.1	9.0	10.9	10.2	81.7	85.5	84.1	77.1	75.7	78.1
Indole-3-acetic acid	41.65	1’486.5	3.7	5.4	3.4	2.2	3.5	3.0	5.4	5.4	6.7	3.7	3.8	4.3	91.8	97.4	95.2	90.9	88.2	83.5
Indoxyl sulfate	131.6	3’113.8	4.6	6.1	4.3	3.8	5.6	3.8	8.3	7.7	7.2	6.5	7.5	6.4	93.3	93.1	92.6	88.0	87.3	87.5
Kynurenic acid	0.93	21.82	5.0	10.9	7.7	5.4	6.2	9.7	5.9	12.1	10.4	6.5	9.3	9.7	97.5	106.1	103.4	94.1	95.0	95.1
Kynurenine	38.29	953.6	3.7	6.2	2.4	2.6	4.0	3.2	5.3	6.2	5.1	2.8	4.0	3.4	95.1	95.3	95.1	90.8	87.1	85.1
N-Acetyl-alanine	15.47	755.3	5.0	4.7	2.5	2.7	3.7	3.5	5.0	4.7	5.7	4.1	5.5	4.7	86.8	82.7	82.4	79.1	81.4	80.5
N-Acetyl-leucine	0.43	42.39	4.9	7.2	9.6	3.2	4.0	3.5	5.2	7.5	11.0	7.8	5.9	4.4	49.7*	67.5*	68.1*	63.8*	77.0	75.9
Nicotinamide	4.00	149.1	3.2	5.8	3.7	4.2	4.2	3.4	7.5	6.7	10.5	9.0	8.7	6.3	95.5	110.8	103.8	101.2	100.4	97.9
Phenylacetyl-glutamine	36.4	3’768	12.3	19.2	10.7	13.0	4.1	6.8	39.2*	46.3*	50.3*	42.7*	35.9*	31.2*	159.2*	158.8*	160.3*	144.8*	107.8	97.8
Serotonin	10.0	249.0	4.2	4.2	3.4	1.9	3.1	2.1	6.9	4.2	4.9	2.1	3.1	2.7	90.2	87.5	82.7	76.2	72.2	71.8
Tryptophan	1’242	37’348	9.2	7.1	7.9	4.0	5.4	6.1	10.6	7.2	9.2	6.2	6.3	6.2	100.2	95.8	95.5	87.8	87.5	87.1

Table S10. Baseline healthy volunteers and CKD patient characteristics. All values are shown as mean (SD). Analytes that were partially validated are indicated with (*). eGFR: estimated glomerular filtration rate computed using the CKD-EPI 2021 formula.

Characteristic	CTRL	CKD3b/4	CKD5
Number, n	19	16	16
Age, years	36.9 (12.6)	65.8 (15.4)	61.1 (20.3)
Male, %	6 (31.5)	13 (81.3)	11 (68.8)
eGFR, mL/min/1.73 m^2	eGFR > 60	44-15	<10 (dialysis 3x/week)
Creatinine, µmol/L	73.16 (12.15)	189.9 (54.0)	804.9 (175.2)
11-Deoxycortisol, ng/mL	0.26 (0.14)	0.42 (0.27)	0.29 (0.32)
3-Hydroxyanthranilic acid*, ng/mL	4.13 (1.4)	7.39 (4.03)	12.06 (5.33)
3-Hydroxykynurenine, ng/mL	8.5 (2.57)	15.59 (4.81)	34.38 (10.3)
5-Hydroxyindoleacetic acid, ng/mL	12.63 (17.58)	31.64 (22.55)	249.77 (101.25)
Androsterone 3α-glucuronide, ng/mL	32.35 (22.83)	75.33 (55.13)	791.89 (778.42)
Anthranilic acid*, ng/mL	1.04 (0.32)	1.18 (0.3)	2.25 (0.71)
Corticosterone, ng/mL	3.73 (3.18)	4.43 (3.86)	2.7 (3.86)
Cortisol, ng/mL	116.06 (37.51)	152.24 (46.08)	110.81 (71.94)
Cortisone, ng/mL	27.01 (5.54)	15.32 (2.31)	8.6 (2.46)
Dehydroisoandrosterone sulfate, ng/mL	1169.9 (710.43)	599.53 (408.73)	656.2 (951.4)
Indole-3-acetic acid, ng/mL	346.21 (124.58)	446.34 (186.09)	929.62 (498.35)
Indoxyl sulfate, ng/mL	1’022.69 (376.15)	3’070.15 (1495.46)	14’366.27 (8842.31)
Kynurenic acid, ng/mL	9.04 (3.05)	26.8 (12.99)	170 (102.48)
Kynurenine, ng/mL	407.83 (112.23)	735.44 (187.33)	834.1 (348.08)
N-Acetyl-alanine, ng/mL	136.75 (17.61)	293.53 (61.75)	644.21 (117.35)
N-Acetyl-leucine*, ng/mL	2.1 (0.66)	5.15 (1.87)	12.07 (5.15)
Nicotinamide, ng/mL	33.56 (15.48)	18.01 (5.79)	40.36 (89.36)
Serotonin, ng/mL	21.64 (15.57)	8.33 (7.25)	5.33 (5.6)
Phenylacetyl-glutamine*, ng/mL	860.61 (688.75)	2’896.86 (933.53)	25’102.81 (8327.8)
Tryptophan, ng/mL	13’828.62 (1909.97)	10’928.86 (2017.56)	4’986.65 (1949.69)

Table S11. Enzyme abbreviation list used in Figure 4 (ordered alphabetically).

Abbreviation	Name
18-Ohase	18-Hydroxylase
3HAO	3-Hydroxyanthranilate oxidase
AADC	Aromatic l-amino acid decarboxylase
ACMSD	2-Amino-3-carboxymuconic semialdehyde decarboxylase
AD	Aldehyde deydrrogenase
AFMID	Arylformamidase
ALDH	Aldehyde dehydrogenase
CYP11B1	11-Beta-hydroxylase
CYP11B2	Aldosterone synthase
CYP17A1	17-Alpha-monooxygenase
CYP21A2	21-monooxygenase
CYPs	Cytochrome P450 enzymes
DHBL	2,5-Dihydroxybenzoate-AMP ligase
HIOMT	Hydroxyindole-O-methyltransferase
HQD	6-Hydroxyquinolinate dioxygenase
HSD11B1	Hydroxysteroid 11-beta dehydrogenase 1
I3PD	Indole-3-pyruvate decarboxylase
IDO	Indoleamine 2,3-dioxygenase
KAT	Kynurenine aminotransferase
KMO	Kynurenine monooxygenase
KYNU	Kynureninase
MAO	Monoamine oxidase
NAMNAT	NAMN adenylyltransferase
NMNAT	Nicotinamide mononucleotide denylyltransferase
NSH	Nonspecific hydroxylation
NUDIX	NAD pyrophosphatase
QDH	Quinolinate hydroxylase
QPRT	Quinolinic acid phosphoribosyltransferase
QS	Quinolinate synthase
SNAT	Serotonin-N-acetyltransferase
SULTs	Sulfotransferases
T5H	Tryptamine 5-hydroxylase
TDO	Tryptophan 2,3-dioxygenase
TnaA	Tryptophanase
TPH	Tryptophan hydroxylase
